# Case report: Persistent hypogammaglobulinemia following hematopoietic stem cell transplantation from a sibling donor with heterozygous RAG1 mutation

**DOI:** 10.3389/fimmu.2026.1798445

**Published:** 2026-04-22

**Authors:** Jing Wu, Huifang Wang, Weijie Chen, Zhiming Hong

**Affiliations:** Pediatric Hematology Department, Quanzhou Maternal and Child Health Hospital (Quanzhou Children’s Hospital), Quanzhou, China

**Keywords:** hematopoietic stem cell transplantation, hypogammaglobulinemia, immune reconstitution, pediatric transplantation, RAG1 mutation

## Abstract

**Introduction:**

Hematopoietic stem cell transplantation (HSCT) is a curative treatment for pediatric hematologic and immunologic disorders, with successful immune reconstitution being critical for long-term survival. We report the unique case of persistent hypogammaglobulinemia following fully matched HSCT from a phenotypically normal sibling donor carrying a heterozygous *RAG1* gene mutation.

**Methods:**

A 13-year-old girl with EBV-associated NK/T-cell lymphoproliferative disorder underwent fully matched sibling HSCT from her mutation-carrying donor. Whole-exome sequencing confirmed the *RAG1* c.994C>T (p.Arg332*) mutation in both donor and recipient. Post-transplant monitoring included serial lymphocyte subset measurements and immunoglobulin level monitoring over a 5-year period.

**Results:**

Despite successful engraftment and quantitative T- and B-cell recovery, the patient developed persistent hypogammaglobulinemia. Serum IgG levels declined to below 4g/L at 4 months post-HSCT and remained persistently below 2g/L from 11 months onwards (with only one measurement of 2.57g/L during this period). IgA levels remained consistently below 0.2g/L, becoming undetectable by 11 months and remaining so. IgM levels dropped below the detection threshold at 2 months and demonstrated only partial recovery by 18 months (peak: 0.29g/L). The patient experienced recurrent respiratory infections, necessitating long-term immunoglobulin replacement therapy.

**Conclusions:**

This case demonstrates that heterozygous variants in genes critical for hematopoietic reconstitution (such as *RAG1* mutations) may still lead to significant immunodeficiency post-transplantation. Our findings strongly suggest that potential donors carrying such mutations require careful evaluation, with non-carrier donors being preferentially selected when available, especially in the context of HSCT.

## 1 Introduction

Hematopoietic stem cell transplantation (HSCT) is an effective treatment for hematological, immunological, and metabolic disorders in children. Successful reconstruction of the hematopoietic system, particularly the immune system, is critical for long-term survival. Post-transplant hypogammaglobulinemia in children can affect up to 77% of patients within the first year (1), and is most commonly transient, which is often associated with delayed B-lymphocyte reconstitution (2, 3). Newly emerging B cells can be detected in the peripheral blood as early as two months after HSCT, with quantitative recovery typically occurring within six to nine months. Immunoglobulin levels subsequently increase; however, complete humoral immune reconstitution may take up to two years (3, 4).

The *RAG1* gene encodes the recombination activating gene 1 (RAG1) protein, which is essential for V(D)J recombination during lymphocyte development (5). Complete *RAG1* deficiency results in severe combined immunodeficiency(SCID), whereas hypomorphic mutations may retain partial protein function, leading to a spectrum of clinical phenotypes characterized by milder or delayed immunodeficiency (6). *RAG1* gene mutations typically follow an autosomal recessive inheritance pattern, and heterozygous mutation carriers are generally phenotypically normal under physiological conditions and thus considered suitable donors for HSCT (7).

However, the transplantation process imposes exceptional regenerative demands on donor-derived hematopoietic progenitors. The long-term immunological consequences of using donors with heterozygous *RAG1* mutations remain poorly understood. No previous cases have reported persistent post-transplant immunodeficiency specifically attributed to heterozygous *RAG1* mutations in donors. Here, we present, to our knowledge, the first reported case of a pediatric patient who developed severe and persistent hypogammaglobulinemia following fully matched sibling HSCT from a phenotypically normal donor carrying a heterozygous *RAG1* mutation, with immunodeficiency persisting up to five years post-transplantation. This case raises important questions about current assumptions regarding donor suitability and highlights the potential need to reconsider donor evaluation protocols. This report details the clinical course and implications.

## 2 Case presentation

### 2.1 Patient information and initial diagnosis

A female patient was admitted at age 5 years 6 months (November 2016) with fever lasting over one month; EBV infection was confirmed by peripheral blood EBV-DNA quantification (1.26 × 10³ copies/mL), which remained detectable at follow-up two months later (1.61 × 10³ copies/mL). At age 8 years, she was diagnosed with chronic active EBV infection (CAEBV), presenting with recurrent fever, hepatosplenomegaly, and hydroa vacciniforme-like skin lesions; EBV-DNA fluctuated between 7.06 × 10² and 1.64 × 10³ copies/mL despite antiviral therapy, with progressive hepatosplenomegaly and generalized lymphadenopathy. EBV serology demonstrated a consistent past-infection pattern (VCA-IgG+/EBNA-IgG+/VCA-IgM−) throughout the disease course. During this period, the patient experienced recurrent fever and skin lesions but did not seek medical attention. In November 2020, at the age of 13 years, EBV-infected lymphocyte subset sorting confirmed T- and NK-cell tropism (B-cell EBV load undetectable), and tissue biopsy with positive EBER *in situ* hybridization established the diagnosis of EBV-positive NK/T-cell LPD, after which the patient underwent allogeneic HSCT in December 2020 at another institution The patient and her elder sister were 10/10 fully human leukocyte antigen (HLA)-matched sibling donor (including complete HLA Class I and Class II matching).

### 2.2 Genetic analysis and donor selection

Whole-exome sequencing conducted prior to transplantation revealed a heterozygous c.994C>T (p. Arg332*) mutation in the *RAG1* gene in both siblings, excluding pathogenic variants in established EBV-LPD susceptibility genes. Family studies demonstrated that the father was a heterozygous carrier, while the mother had a wild-type genotype. Pre-transplant immunological evaluation of the sister and father confirmed normal immunoglobulin levels and lymphocyte subsets in both individuals, consistent with asymptomatic heterozygous carrier status under autosomal recessive (AR) inheritance. Although the *RAG1* c.994C>T (p.Arg332*) variant was classified as ‘Pathogenic (P)’ by ACMG criteria—a classification applied to the biallelic disease state—heterozygous carriers are not expected to manifest clinical disease. Given the urgency of HSCT due to progressive EBV-positive NK/T-cell LPD and considering the sister was the only available 10/10 HLA-matched donor, she was selected as the donor. Immunological data post-transplant for the patient, sister, and father are presented in **Table 1**.

**Table 1:** Immunoglobulin levels and lymphocyte subset of the patient, sister, and father.

Subset	Ig levels (g/L)			Lymphocyte subset cells/µl (%)				
	IgG	IgA	IgM	CD3+	CD3+CD4+	CD3+CD8+	CD19+	NK
patient	3.42	<0.06	0.33	1936 (80)	678 (28)	1113 (46)	339 (14)	97 (4)
sister	10.8	1.26	0.88	1725 (69)	875 (35)	750 (30)	375 (15)	350 (14)
father	13.0	1.55	0.6	1960 (70)	868 (31)	1064 (38)	336 (12)	476 (17)

Data for all three individuals were obtained on the same date (48 months post-HSCT for the patient; immediately prior to IVIG infusion), following the initiation of regular IVIG replacement therapy. Ig, immunoglobulin.

### 2.3 Transplantation procedure and early outcomes

Following a myeloablative conditioning regimen comprising busulfan, cyclophosphamide, and antithymocyte globulin (Bu/Cy+ATG, without total body irradiation), the patient received peripheral blood stem cell infusion from her sister in December 2020. HSCT was performed within one month of admission, during which time no disease-specific cytotoxic chemotherapy or IVIG was administered. Pre-transplant immunoglobulin levels were within normal limits (IgG 14.2 g/L, IgA 1.11 g/L, IgM 0.18 g/L), and no IVIG was administered prior to or during transplantation. Neutrophil engraftment (>0.5×10^9^/L) was achieved on day +11 post-transplantation, and platelet engraftment (>20×10^9^/L) on day +12. Cyclosporine was administered as GVHD prophylaxis until six months post-HSCT. The patient did not receive rituximab therapy throughout the entire treatment course, and no acute/chronic GVHD or viral reactivation was observed during the entire follow-up period.

### 2.4 Immune reconstitution and long-term outcomes

**Figure 1:**
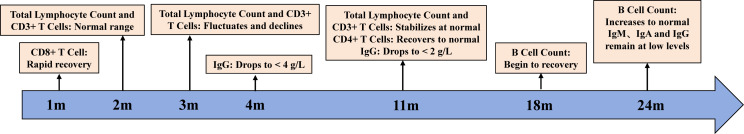
Immune reconstitution and clinical course post-hematopoietic stem cell transplantation (HSCT).

**Figure 2:**
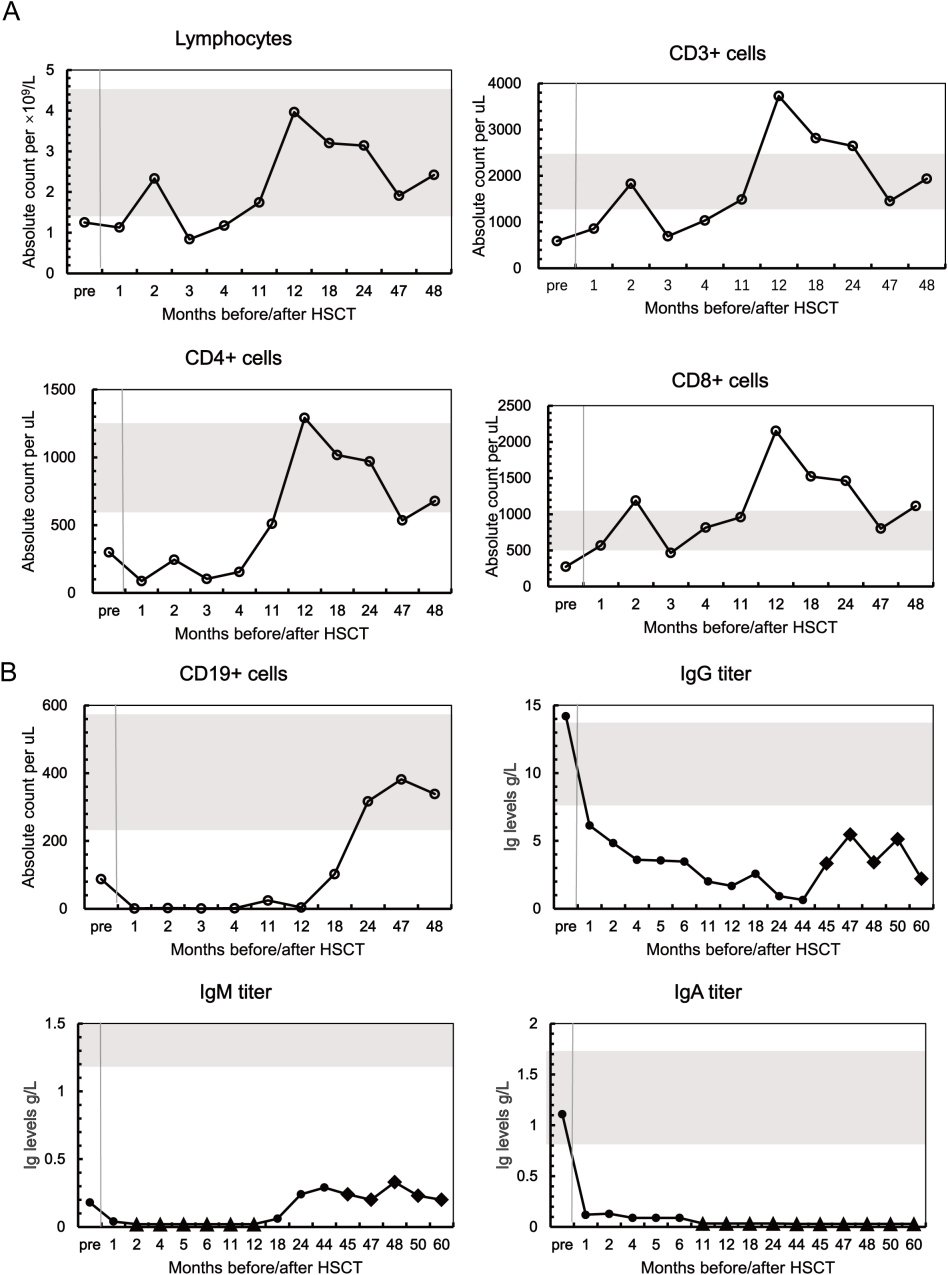
Cellular and humoral immune recovery post-HSCT. **(A)** Lymphocyte counts and T-cell levels in the patient compared to the age-matched normal range (gray bars). **(B)** B-cell and serum immunoglobulin levels compared with age-matched reference ranges (gray bars). •, baseline values; ♦, post-IVIG intervention, all serum immunoglobulin measurements were obtained as pre-infusion trough levels; ▴, instrument detection limits.

Initial immune reconstitution demonstrated complex dynamic changes (**Figure 1**). The patient’s total lymphocyte count recovered to normal range at 2 months post-transplantation, but declined again at 3 months, then increased and approached normal levels at 4 months. CD8+ T-cell counts rapidly recovered within 1 month. However, CD3+ T-cell counts exhibited an fluctuating recovery pattern, rising to normal levels at 2 months but subsequently declining, and did not stabilize within normal range until 11 months post-transplantation. CD4+ T-cell count recovery was relatively delayed, reaching normal levels only after 12 months (**Figure 2A**).

During the early post-transplant phase, B-cell counts remained below 102 cells/ul, far below the normal reference range (247–578 cells/µL). B-cell counts remained persistently low in the early post-transplant period and did not show numerical increase and recovery to normal levels until 24 months; however, immunoglobulin levels failed to achieve corresponding recovery (**Figure 2B**). Serum IgG levels declined to below 4 g/L at 4 months post-HSCT and remained persistently below 2 g/L from 11 months onwards (with only one measurement of 2.57 g/L during this period). IgA levels remained consistently below 0.2 g/L, becoming undetectable by 11 months and remaining so. IgM levels dropped below the detection threshold at 2 months and demonstrated only partial recovery by 18 months (peak: 0.29 g/L).

### 2.5 Clinical complications and management

At 18 months post-transplantation, bone marrow examination including cytology and immunophenotyping was performed and revealed no abnormalities; however, this normal cellular reconstitution contrasted sharply with functional deficits. Recurrent respiratory infections emerged at approximately 18 months post-transplantation, initially managed with oral antibiotics. Secondary immunoglobulin loss was excluded on clinical grounds: serum albumin and renal function remained within normal limits throughout follow-up, urinary protein was consistently negative, and no clinical manifestations of renal or gastrointestinal disease were identified. The clinical situation deteriorated over time: at approximately four years post-transplantation, she was hospitalized for pneumonia and initiated on regular IVIG replacement therapy (0.4 g/kg every 4 weeks). Serum IgG rose to 3.33 g/L after the initial IVIG dose, peaked at 5.47 g/L following subsequent regular infusions, and declined to 3.42 g/L four weeks later. The most recent pre-infusion IgG level was 2.22 g/L, indicating only transient IgG improvement without sustained endogenous recovery. (**Figure 2B**).

### 2.6 Current status

During five years of follow-up, this case demonstrates a unique pediatric presentation of persistent hypogammaglobulinemia following fully matched HSCT from a sibling donor carrying a heterozygous *RAG1* mutation. Despite adequate quantitative T- and B-cell reconstitution, humoral immunity remained significantly impaired, characterized by persistently low levels of IgG, IgA, and IgM, suggesting profound functional B-cell defects that necessitated long-term immunoglobulin replacement therapy.

### 2.7 Ethics statement

This case report was conducted in accordance with the Declaration of Helsinki and approved by the Institutional Review Board of Quanzhou Maternal and Child Health Hospital (Quanzhou Children’s Hospital) (Ethics approval No. 2026-5). Written informed consent was obtained from the patient’s legal guardian for the publication of this case report.

## 3 Discuss

This case report presents the first pediatric patient to develop persistent hypogammaglobulinemia after receiving a fully matched sibling donor transplant from a carrier of a heterozygous *RAG1* mutation. Despite adequate reconstitution of T and B cell numbers, humoral immunity remained significantly impaired, characterized by persistently low IgG, IgA, and IgM levels, indicating a functional B cell defect. To our knowledge, this is the first report linking a donor heterozygous *RAG1* mutation with persistent post-transplant immune deficiency in the recipient. While we cannot draw definitive conclusions from a single case, this observation may warrant reconsideration of current donor evaluation standards and underscores the need for further investigation.

In pediatric patients, post-transplant hypogammaglobulinemia is typically associated with delayed B cell recovery, total body irradiation (TBI), or graft-versus-host disease (GVHD) (8). The literature describes a case of persistent hypogammaglobulinemia lasting up to seven years after allogeneic hematopoietic stem cell transplantation, with normal peripheral B cell counts; further analysis suggested this may be related to terminal B-cell differentiation defects caused by HLA class II mismatch (9). The use of rituximab after transplantation can also lead to recovery of B cell numbers while function remains impaired (10). However, in this case, the donor was fully HLA-matched, no TBI or rituximab was used, and there was no occurrence of acute or chronic GVHD, effectively ruling out these common causes. In addition, secondary immunoglobulin loss was considered and excluded, with no clinical or laboratory evidence of renal or gastrointestinal disease throughout follow-up.

After exclusion of the traditional pathogenic factors outlined above, the heterozygous *RAG1* c.994C>T (p.Arg332*) mutation emerges as the most plausible underlying cause of the observed immunodeficiency. RAG1 plays a critical role in V(D)J recombination during lymphocyte development, and mutations in this gene are associated with broad phenotypic heterogeneity ranging from severe combined immunodeficiency to milder, atypical presentations (11). Crucially, *RAG1* haploinsufficiency may remain clinically silent in donors under normal physiological conditions—as confirmed by the intact immune profiles of the donor sister and father—yet become functionally significant under the heightened demands of *de novo* lymphoid reconstitution following HSCT. The p.Arg332* mutation is expected to produce a truncated, nonfunctional RAG1 protein (12); furthermore, Abraham et al. demonstrated that comparable heterozygous *RAG1* frameshift mutations retain only 2.67% ± 0.58% of normal recombination activity (13), supporting the biological plausibility of meaningful impairment from a single defective allele. Consistent with this mechanism, a striking quantitative–functional dissociation was observed: although CD19+ B cells recovered to 317 cells/µL (10.1% of lymphocytes) by 24 months post-transplantation—within the age-matched reference range—IgG remained at 0.64 g/L at 44 months and IgA was persistently undetectable throughout five years of follow-up, indicating a cell-intrinsic defect in late B-cell differentiation and class-switch recombination rather than a failure of B-cell production. Furthermore, the transplantation process itself may further exacerbate the functional impact of such genetic defects through epigenetic modifications, oxidative stress, and altered cytokine environments, thereby compounding impairment of normal immune reconstitution pathways.

Several limitations of this report merit acknowledgment. As a single case report, our findings require validation in larger cohorts of patients who have undergone HSCT from heterozygous *RAG1* carrier donors. Direct functional studies—including V(D)J recombination activity assays and *in vitro* B-cell differentiation assays—were not performed, nor was detailed B-cell subset phenotyping, as the patient’s family declined further investigation during follow-up; consequently, the functional significance of the p.Arg332* mutation under HSCT-driven reconstitution remains mechanistically plausible but unproven. The possible contribution of other genetic or environmental factors also cannot be fully excluded. Going forward, we plan longitudinal monitoring of IgG levels in the patient’s sister and father and may pursue additional functional B-cell assessments to better delineate the pathogenicity of the heterozygous *RAG1* state.

In summary, we report the first case of persistent hypogammaglobulinemia following transplantation from a donor carrying a heterozygous *RAG1* mutation. This observation raises the possibility that heterozygous variants in genes critical for lymphoid reconstitution may carry post-transplant immunological consequences not apparent under normal physiological conditions. Therefore, we suggest that clinicians be aware of this potential risk when evaluating donors carrying heterozygous *RAG1* variants, particularly when alternative non-carrier donors are available. However, conclusions from a single case are insufficient to challenge current donor selection standards or justify systematic expansion of genetic screening protocols for heterozygous variants. Further investigation in larger cohorts is essential before such policy changes are implemented.

## Data Availability

The genetic variant identified in this study has been deposited in the ClinVar public database and is accessible under accession number SCV007537781 (https://www.ncbi.nlm.nih.gov/clinvar/variation/649706/?term=%22SCV007537781%22).
